# Nonnegative Mixed-Norm Convex Optimization for Mitotic Cell Detection in Phase Contrast Microscopy

**DOI:** 10.1155/2013/176272

**Published:** 2013-11-19

**Authors:** Anan Liu, Tong Hao, Zan Gao, Yuting Su, Zhaoxuan Yang

**Affiliations:** ^1^School of Electronic Information Engineering, Tianjin University, Tianjin 300072, China; ^2^College of Life Sciences, Tianjin Normal University, Tianjin 300387, China; ^3^Key Laboratory of Systems Bioengineering, Ministry of Education, Department of Pharmaceutical Engineering, School of Chemical Engineering and Technology, Tianjin University, Tianjin 300072, China; ^4^Key Laboratory of Computer Vision and System, Tianjin University of Technology, Tianjin 300384, China

## Abstract

This paper proposes a nonnegative mix-norm convex optimization method for mitotic cell detection. First, we apply an imaging model-based microscopy image segmentation method that exploits phase contrast optics to extract mitotic candidates in the input images. Then, a convex objective function regularized by mix-norm with nonnegative constraint is proposed to induce sparsity and consistence for discriminative representation of deformable objects in a sparse representation scheme. At last, a Support Vector Machine classifier is utilized for mitotic cell modeling and detection. This method can overcome the difficulty in feature formulation for deformable objects and is independent of tracking or temporal inference model. The comparison experiments demonstrate that the proposed method can produce competing results with the state-of-the-art methods.

## 1. Introduction

Measurement of the proliferative behaviors of cells *in vitro* is important to many biomedical applications, such as drug discovery, stem cell manufacturing, and tissue engineering. Recently, the need for extended-time observation and the proliferation of high-throughput imaging have made automatic mitotic cell detection mandatory.

The state-of-the-art methods for this task generally fall into two categories. (1)   *Spatial feature-based method*: this kind of methods detects mitotic cells directly in an image depending on spatial visual characteristics. Liu et al. [1] considered mitotic cell as a special visual pattern and train a Support Vector Machine classifier with region features for identification. Li et al. [2] extracted volumetric Haar-like features and implemented a cascade framework to classify spatiotemporal sliding windows of an image sequence. Since the current low level visual features usually have low discrimination for nonrigid and deformable objects, this kind of methods always achieves unsatisfactory performances. (2) *Sequential feature-based method*: this kind of methods usually implement object tracking or temporal inference models to leverage sequential features for decision. Yang et al. [3] extracted individual cell trajectories by cell tracking and identified mitoses with the dynamic features of the mother and daughter cells during mitosis progression. To handle the difficulty by cell tracking, temporal inference models are implemented to leverage the temporal context for mitosis event recognition. Gallardo et al. [4] trained a hidden Markov model for mitosis recognition with cell shape and appearance dynamics. Liu et al. [5] applied a *hidden-state conditional random field* to learn the sequential structure of mitosis progression. Liang et al. [6] implemented a *conditional random field* model [7] to further localize different mitotic phases based on the visual features of the nuclei.

**Figure 1:**
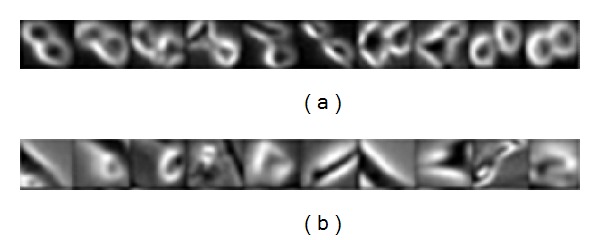
(a) Mitotic cell samples; (b) Nonmitotic cell samples.

Although much work has been done on this task, there still exist several limitations. On one hand, the state-of-the-art visual features can not discriminatively represent mitotic cells with irregular appearance changes as shown in Figure 1 and therefore the *spatial feature-based method* only has relatively low generalization. On the other hand, the *sequential feature-based method* involves temporal inference model to take advantage of temporal context. Learning the complicated transition among multiple states will induce high computational complexity and make the system far from the requirement of real-time mitotic cell recognition for biological analysis.

To tackle this challenging task, we propose a nonnegative mix-norm convex optimization method for mitotic cell detection. First, we apply an imaging model-based microscopy image segmentation method that exploits phase contrast optics to extract mitotic candidates in the input images. Then, a convex objective function regularized by mix-norm with nonnegative constraint is proposed to induce sparsity and consistence for discriminative representation of deformable objects. At last, a Support Vector Machine classifier is utilized for model learning and detection on the extracted candidate regions. The main contribution lies in two folds. (1) This method can overcome the difficulty in feature formulation for deformable objects. (2) It is independent of tracking or temporal inference model and can greatly reduce the computational complexity.

The rest of paper is structured as follows. In Section 2, we briefly introduce the systematic workflow. Then, the mitotic cell representation is illustrated in Section 3. The experimental method and results will be detailed in Sections 4 and 5. At last, conclusion is presented.

## 2. System Overview

The proposed method was designed to automatically identify mitotic cell in phase contrast microscope images. To achieve this goal, the method proceeds through tree consecutive steps.

*(i) Mitotic Candidate Extraction*. This step aims to extract candidate regions, *C* = {*C*_*i*_}_*i*=1_^*N*^(*C*_*i*_ ∈ *R* ^*m*×*n*^, *m*  and  *n*  separately mean the width and height of one region), that potentially contain mitotic cells from the original image, while eliminating most background regions to reduce the search space for refinement. We adopted the imaging model-based microscopy image segmentation method proposed in our previous work [1, 8] to detect mitotic cells in the input sequence. Since this step is not the main focus of this paper, we only briefly introduce it as follows. Please refer to [1, 8] for more details.

Under a positive phase contrast microscope, adherent stem cells growing in culture appear as dark objects surrounded by bright halos. We have proposed an imaging model-based microscopy image segmentation method [1, 8] to restore the ideal image in which pixel values are positive inside cell regions while being uniformly zero in the background. The objective function is

(1) O(f)=||Pf−g||22+wsmoothf⊤Lf+wsparse||Df||1,subject  to  fk≥0,  ∀k.

During mitosis, stem cells usually appear as drastically intensified halo artifacts, which often completely immerse the cell while their volume reaches minimum. By comparing these two phenomena, we found that the visual pattern of the mitotic region in the inverted phase contrast microscopy, −*f*, is similar to the visual appearance of the normal cell region in the original microscopy,  *f*. Therefore, we can selectively enhance only mitotic regions by modifying $\overset{-}{f}=-f$ and formulate the objective function equation

(2) O(f−)=||Pf−+g||22+wsmoothf−⊤Lf−+wsparse||Df−||1,subject  to  f−k≥0,  ∀k,

where **g** and $\overset{-}{f}$ are *N*-dimensional vectorized representations of the *observed* image *g*(*i*, *j*) and the *artifact-free* image *f*(*i*, *j*) with only mitotic cells, respectively, with *N* being the number of pixels in the image; **L** and **D** are, respectively, a Laplacian matrix and a diagonal matrix defining local smoothness and sparseness with corresponding weights *w*_smooth_ and *w*_sparse_ [8, 9]. The similarity-based Laplacian matrix **L** is defined by **L** = **D** − **W**. **W** is a symmetric matrix whose off-diagonal elements are defined a *W*_*mn*_ = *e* ^−(*g*_*m*_−*g*_*n*_)^2^/*σ*_1_^ where *g*_*m*_ and *g*_*n*_ denote intensities of neighboring pixels *m* and *n*,*σ*_1_ is the mean of all possible (*g*_*m*_ − *g*_*n*_)^2^ in the image, and **D** is a diagonal degree matrix where *D*_*mm*_ = ∑_*n*_ *W*_*mn*_. ||·||_*p*_ denotes the *L* ^*p*^ norm; the nonnegativity constraint on **f** enforces the assumption that the cell-induced phase shifts to be restored are unidirectional; hence the restored pixel values are positive inside cell regions while being uniformly zero in the background; **P** is a *N* × *N* matrix such that the *k*th element of the vector $P\overset{-}{f}$ can be computed by the convolution between $\overset{-}{f}$ and the discretized point spread function $\text{PSF}(u,v)=\delta (u,v)-\text{airy}(\sqrt{u^{2}+v^{2}})$, where airy(·) is an obscured Airy pattern [10–12].

The image $\overset{-}{f}$ can be obtained by minimizing $O(\overset{-}{f})$ using iteratively reweighted nonnegative multiplicative update [9]. Samples are shown in Figure 3.

*(ii) Mitotic Cell Representation.* This step aims to represent *C*_*i*_ with a high level feature vector which directly denotes the similarity between one sample and the bases of the dictionary in the sparse representation scheme. Given a training set consisting of positive and negative mitotic cell regions and the corresponding low level image feature set *X* = {*X*_*i*_}_*i*=1_^*N*^ (*X*_*i*_ ∈ *R* ^*d*×1^), this step means to decompose *X*_*i*_ over a dictionary Φ (Φ = {*ϕ*_*i*_}_*i*=1_^*M*^, *ϕ*_*i*_ ∈ *R* ^*d*×1^ is basis denoting a classic visual pattern) such that *X*_*i*_ = Φ · *w*_*i*_ + *r*_*i*_ where *w*_*i*_ ∈ *R* ^*M*×1^ is a sparse vector and *r*_*i*_ ∈ *R* ^*d*×1^ is the residual. Since *w*_*i*_ denotes the correlation between *X*_*i*_ and each basis, it can be utilized as the feature representation of one sample. We proposed a convex objective function regularized by mix-norm with nonnegative constraints to simultaneously obtain the optimal W* = {*w*_i_*}_*i*=1_^*N*^ and Φ*. This method will be detailed in Section 3.

*(iii) Mitotic Cell Detection.* Under the sparse representation scheme, each test candidate region with *X*_*t*_ as its low level image feature can be represented as a linear combination of bases in Φ* by coefficient *w*_*t*_. Therefore,  *w*_*t*_  also reflects the relationship between  *X*_*t*_  and bases and can be utilized as the characteristic representation for test. In our work, an SVM classifier was trained with the high level feature set W* = {*w*_i_*}_*i*=1_^*N*^. Then it is utilized to predict each test sample,  *w*_*t*_, as mitotic cell or not. SVM is a supervised binary classifier that constructs a linear decision boundary or a hyperplane to optimally separate two classes. Literature show that SVM usually has high generalization ability especially when only small amount of training data is available [14].

## 3. Mitotic Cell Representation

### 3.1. Problem Formulation

For the representation of one mitotic cell, *X*_*i*_, we designed the objective function as follows

(3) Obj(wi,γ1,γ2)=||Xi−Φ·wi||22+γ1Sparsity(wi)+γ2Consistence(wi),subject  to  wi≥0,

where ||·||_2_ means a *L*_2_ norm; the relative importance of three terms is controlled by the positive weights *γ*_1_ and *γ*_2_; the nonnegative constrain (*w*_*i*_ ≥ 0) is imposed because  *w*_*i*_  represents the similarity between one sample and bases. The optimal decomposed coefficient *w*_i_* can be achieved by solving *w*_i_* = argmin⁡_*w*_*i*__Obj(*w*_*i*_, *γ*_1_, *γ*_2_). The objective function in (3) consists of three parts.

*(i) Fidelity.* The first term penalizes the sum-of-squares difference between the reconstructed and original sample. Assuming that there are enough training samples of mitotic cell regions so that the dictionary Φ consisting of all these samples is overcomplete, it is obvious that a new mitotic cell image can be faithfully represented only by the linear combination of mitotic bases. However, it is impossible to enumerate all mitotic cases for training set in reality. The sparse coding in this way will be rather unstable. A feasible compensation is to utilize the samples of nonmitotic cell regions. By tuning *w*_*i*_, the negative samples might by very helpful for reconstruction when there are only limited mitotic bases. Therefore, the *Fidelity* term will utilize both mitotic and nonmitotic samples for reconstruction. 

*(ii) Sparsity.* In the sparse coding scheme, it is usually expected that a mitotic sample should be reconstructed with both low residual and few mitotic bases. Although a nonmitotic sample can also be reconstructed with the same dictionary and the acceptable residual, it will leverage lots of bases for compensation and result in a dense  *w*_*i*_. Lasso penalty is well known to impose sparsity for decomposition [15]. Therefore, ||*w*_*i*_||_1_ (*L*_1_ norm of *w*_*i*_) is implemented for *Sparsity* term. 

*(iii) Consistence.* In the framework of sparse representation, overcomplete dictionary always exists and consequently the dictionary would be redundant. It is known that to induce sparsity the lasso tends to select only one basis from the group of bases which have high correlations in between and consequently lead to the nonunique solutions. To handle this problem, in the objective function equation (3) for mitotic cell representation, we impose the ridge penalty ||*w*_*i*_||_2_ (*L*_2_ norm of *w*_*i*_) for *Consistence* term. Zou and Hastie [16] mathematically demonstrated that strict convexity could guarantee the consistence in the extreme situation with identical bases. Since the linear combination of the lasso and ridge penalties is strictly convex, the regularizations of the objective function equation (4) can benefit from preserving the consistence. Furthermore, Zou and Hastie [16] derived the upper bound of the difference between the coefficients of two different bases to quantitatively describe the consistence effect by the elastic net penalty [16, Theorem  1]. Following Theorem 1, the difference between the coefficients of two bases is almost 0 if these bases are highly correlated. Therefore, the *Consistence* term can theoretically avoid the nonunique solution when the dictionary is redundant.

In this way, the proposed convex objective function regularized by mix-norm with nonnegative constraint can be formulated as follows:

(4) Obj(wi,γ1,γ2)=||Xi−Φ·wi||22+γ1||wi||1+γ2||wi||2,subject  to  wi≥0.

### 3.2. Optimization

 Given a training set of  *N*  samples, *X* = {*X*_*i*_}_*i*=1_^*N*^, where *X*_*i*_ denotes the extracted visual feature of each training candidate, there exists a latent dictionary of bases where each basis characterizes a special visual pattern of mitotic cell region or nonmitotic cell region such that a new image can be sparsely reconstructed with respect to this dictionary. Therefore, the goal of optimization of the objective function is to discover the optimal dictionary Φ* and reconstruction coefficients *w*_i_* for the corresponding *X*_*i*_. This task can be achieved by solving the optimization problem following:

(5) (Φ∗,W∗)=argmin⁡Φ∈ℂ,W∈ℝM×N∑i=1N(||Xi−Φ·wi||22+γ1||wi||1+γ2||wi||22),subject  to  wi≥0,

where *W* = {*w*_*i*_}_*i*=1_^*N*^ and the convex set *ℂ* = {Φ ∈ ℝ^*d*×*M*^, s.t., for  all  *i*, ||*ϕ*_*i*_||_2_^2^ ≤ 1}. Since the optimization problem above is not convex with respect to both Φ and *W*, we follow the coordinate decent framework and propose the Iterative Updating method and summarize it in Algorithm 1, which is well tailored from the online learning algorithm [17]. Assuming the training set consisting of i.i.d. samples from a distribution *p*(*x*), the proposed algorithm randomly draws one sample *x*_*t*_ at a time and alternates the sparse coding step for computing *w* ^*t*^ of *x*_*t*_ over the dictionary Φ^*t*−1^ obtained at the previous iteration and the dictionary updating step for computing the new dictionary Φ^*t*^ with respect to *w* ^*t*^. The two main components of the method are, respectively, presented below.

#### 3.2.1. Sparse Coding

 Given the obtained dictionary in (*t* − 1)th iteration, Φ^*t*−1^, the coefficient of *X*_*t*_ in  *t*th  iteration, *w* ^*t*^, for updating, is independent of others. Therefore, we can optimize them independently as follows:

(6) wt=argmin⁡w∈ℝM×1(||Xt−Φt−1·w||22+γ1||w||1+γ2||w||22),subject  to  w≥0.

For this convex objective function regularized by L1/L2 mix-norm with nonnegative constraint, we adopt the linear-time projection method on the L1/L2 mix-norm regularization [17]. To fulfill the nonnegative constraint, we only keep the members of  *w*  which are greater than 0 and set the others with 0 in each interaction.

With the optimal dictionary Φ*, the optimal decomposition coefficients W* can be achieved by

(7) W∗=argmin⁡W∈ℝM×N∑i=1N(||Xi−Φ∗·wi||22+γ1||wi||1+γ2||wi||22),subject  to  wi≥0.

#### 3.2.2. Dictionary Updating

During the  *t*th  iteration, the algorithm will aggregate the previous information computed from loop 1 to loop  *t*  for dictionary updating. Given the obtained *w* ^*t*^ for *X*_*t*_ in  *t*th  iteration, the expected dictionary in  *t*th  iteration, Φ^*t*^, can be obtained by minimizing the average error over all *t* iterations. (Here 1 ≤ *t* ≤ *T*_max⁡_ (the maximum iteration) and *T*_max⁡_ is independent of the sample number, *N*, of the training set.) Therefore, Φ^*t*^ can be optimized by

(8) $$\begin{array}{c} \Phi^{t}=\arg\underset{\Phi \in \mathbb{C}}{\min}\frac{1}{t}\sum_{i=1}^{t}{||X_{i}-\Phi \cdot w^{i}||}_{2}^{2}. \end{array}$$

The constraint ||*ϕ*_*i*_^*t*^||_2_^2^ ≤ 1(∀*i*) is implemented to avoid basis *ϕ*_*i*_^*t*^ being arbitrarily large, which would result in arbitrarily small value of *w*. Equation (8) can be solved as illustrated in Algorithm 2.

## 4. Experimental Method

### 4.1. Data

 Two challenging phase contrast image sequences of C3H10T1/2 mouse mesenchymal stem cell populations (American Type Culture Collection, Manassas, VA) were acquired, each containing 1436 images. The growing environment consists of Dulbecco's Modified Eagle's Media (DMEM; Invitrogen, Carlsbad, CA), 10% fetal bovine serum (Invitrogen, Carlsbad, CA) and 1% penicillin streptomycin (PS; Invitrogen, Carlsbad, CA). The cells were observed during growth under a Zeiss Axiovert 135TV inverted microscope with a 5x, 0.15 N.A. objective and phase contrast optics. Time-lapse image acquisition was performed every 5 minutes using a 12-bit Qimaging Retiga EXi Fast 1394 CCD camera at 500 ms exposure with a gain of 1.01. Each image consists of 1392 × 1040 pixels with a resolution of 19 *μ*m/pixel.

After image acquisition, the bounding box of each mitotic cell was manually annotated by an expert biologist using a labeling tool with a graphical user interface and then resized to an image patch of 25 × 25  pixels. The nonmitotic candidates in the same size were automatically and randomly selected. Totally, the training set consists of 222 positive samples and 783 negative samples from one image sequence and the test set consists of 359 positive samples and 1267 negative samples from the other.

### 4.2. Experiments

 Any state-of-the-art visual feature can be utilized for low level image representation. To demonstrate that the proposed method does not need specific object-dependent visual feature formulation with high discriminative capability, we extracted the pixel-wise intensity feature as well as three representative visual features for comparison. Pixel-wise intensity feature (Raw) represents the global intensity distribution of one image and implicitly contains appearance characteristics. This feature is formed by concatenating each pixel intensity in raster order [9]. Histogram of Oriented Gradients (HoG), GIST, and Scale Invariant Feature Transform (SIFT) are widely utilized to represent the shape characteristic, local structural information, and local visual saliency, respectively. Due to the limited space, please refer to [18–20] for more details.

For dictionary construction, we need to discover which visual feature and what configuration of *γ*_1_ and *γ*_2_ are the best combination. Specifically, by fixing *γ*_1_ and *γ*_2_, we can compare the performances of the learned SVM models with respect to four kinds of visual features. Moreover, by fixing the visual feature, we can compare the performances of the configurations of *γ*_1_ and *γ*_2_ by tuning both within  [10^−4^, 10^−1^].

To demonstrate the superiority of the proposed method for mitotic cell detection, we compared its performance against the spatial feature-based method [1]. Both methods only use spatial visual features for recognition and can form a fair comparison. We utilized the decomposed coefficients by the proposed method with respect to each visual feature and the best corresponding configurations of *γ*_1_ and *γ*_2_ to train SVM models separately. Comparatively, we directly used each kind of visual features extracted from the same training set to train a corresponding SVM model. We compared the performances with the same test set. Moreover, we compared the proposed method to the recently popular sequential feature-based methods, including Hidden Markov Model-based method (HMM) [4], Conditional Random Field-based method (CRF) [6], Hidden-state Conditional Random Field-based method (HCRF) [5], and the most recently proposed EDCRF-based method (EDCRF) [13].

To evaluate the performance of mitotic cell detection, we examined four different outcomes by the proposed method, true positive (TP), false negative (FN), false positive (FP), and true negative (TN). Precision (TP/(TP + FP)), Recall (TP/(TP + FN)), and the *F*_1_ score ((2 × Precision × Recall)/(Precision + Recall), representing the overall performance of both)) are used as quantitative metrics to evaluate the performance of mitotic cell recognition. Accuracy ((TP + TN)/(TP + FN + FP + TN)) is utilized to evaluate the overall performance of both mitotic and nonmitotic cell recognition.

## 5. Experimental Results and Discussion

### 5.1. Mitotic Candidate Extraction

 The proposed mitotic candidate extraction method achieved 100% recall and 40% precision on the test sequence as compared to the ground truth. Some examples of the extracted candidates are shown on the right of Figure 3. It is intuitive that our method eliminates most of the surrounding background of mitotic cells, keeping only the essential visual patterns for feature extraction. This helps improve the performance of mitotic cell detection as presented next.

### 5.2. Mitotic Cell Detection

**Figure 2:**
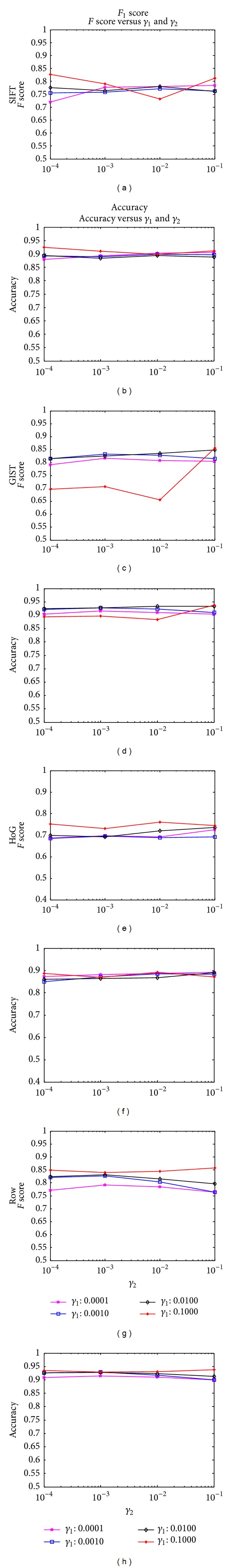
Performance comparison for dictionary learning with respect to four kinds of visual features and different configurations of  *γ*_1_  and  *γ*_2_.

 The performances of different dictionary learning strategies with respect to four visual features and different configurations of *γ*_1_ and *γ*_2_ are shown in Figure 2. With the comparison in each row, we can achieve the best *F*_1_ score and accuracy when both *γ*_1_ and *γ*_2_ were 0.1 and the visual feature was fixed. It is implied that the stronger sparsity and consistence effects can benefit the decomposed coefficients for model learning. In our experiment, the maximum standard deviation (MSD) of *F*_1_ score by fixing *γ*_1_/*γ*_2_ and tuning *γ*_2_/*γ*_1_ is 0.044 (except the special case of 0.086 when using GIST and *γ*_1_ = 0.1 shown in Figure 2(c)) and the MSD of accuracy is 0.019. These results show that the proposed method has strong robustness with respect to different visual features and a broad range of parameters.

**Figure 3:**
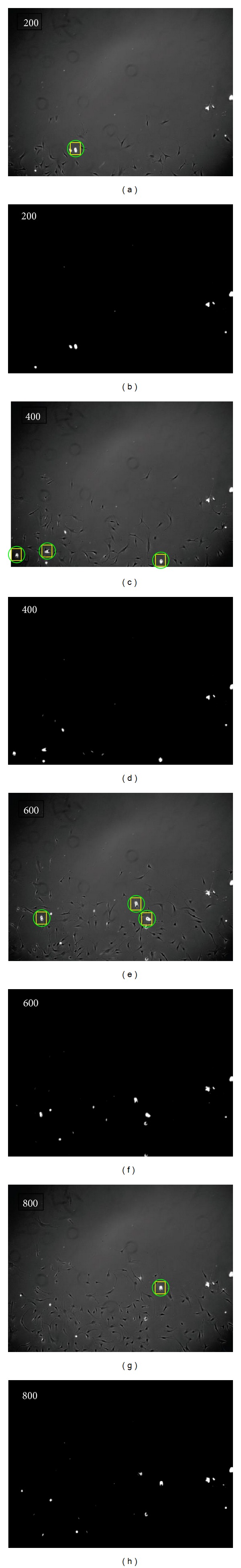
Sample images of mitotic cell detection on C3H10 image sequence (the number on the left top corner means the frame number in the image sequence). Left: original images with the ground truth (yellow rectangle) and detected regions (green circle). Right: mitotic candidate detection results by the imaging model-based microscopy image segmentation method. The white regions denote the detected mitotic candidates for further identification.

**Figure 4:**
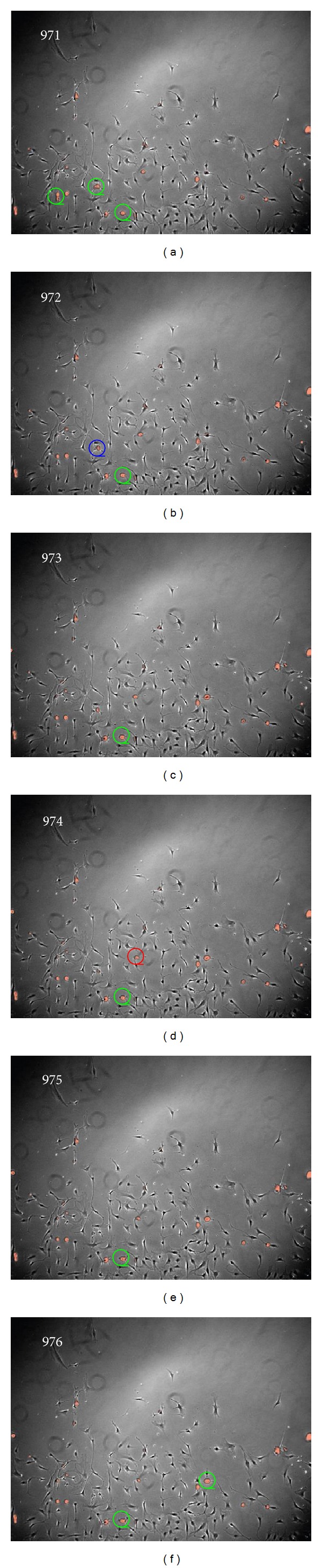
Sample images of mitotic cell detection on C3H10 image sequence with high confluency (the number on the left top corner means the frame number in the image sequence). The red regions overlaying the original images denote the detected mitotic candidates by the imaging model-based microscopy segmentation method. Green, blue, and red circles, respectively, denote true positive, false negative, and false positive.

The best performances with respect to different visual features when  *γ*_1_  and  *γ*_2_  are both fixed with 0.1 were compared to decide which feature is the best for representation. From the left side of Table 1, it is obvious that the dictionary learned with Raw consistently outperforms others. It implies that it is not necessary to develop special visual feature to overcome the variance of rotation, scale, shape, and so on for mitotic cell detection although its appearance may changes irregularly. Comparatively, the decomposed coefficients of one sample can explicitly reflect its correlation with bases and this relationship can be achieved stably by the optimization method regularized by L1/L2 mix-norm with nonnegative constraint even though we did not explicitly align mitotic samples as we usually do for face recognition. Therefore, the proposed method can avoid the nontrivial task of feature extraction for deformable object recognition. The left column of Figure 3 shows the samples of the final mitotic cell detection on the frames with increasing confluency in one C3H10 image sequence. Especially, when cell density got much higher as shown in Figure 4, the proposed method can still effectively identify mitotic cell with the discriminative and robust high level feature stably produced by the convex optimization regularized by L1/L2 mix-norm with nonnegative constraint although one false positive and one false negative cases occur.

### 5.3. Comparison

 The performance comparison between the proposed method and the spatial saliency-based method (SSM) [1] is shown in Table 1. It is obvious that the proposed method can consistently outperform the other in terms of *F*_1_ score and accuracy with respect to any visual feature. Especially, we can achieve the best performance (*F*_1_ = 85.7% and accuracy = 93.9%) when Raw was selected and both  *γ*_1_  and  *γ*_2_  were 0.1, which is competitive to the performance of GIST by both methods. However, the formulation of GIST would cost much higher computation [20] compared to the formulation of Raw. To our surprise, SIFT feature works worse than both Raw and GIST. It is explainable that the substantial and irregular appearance changes can not be preserved simply by SIFT formulation. It is expected that HoG works worst because it mainly represents the shape feature and is not suitable for deformable object representation.

The advanced comparison to the temporal context-based methods with Raw as low level feature is summarized in Table 2. The proposed method achieved the precision of 88.0% and the recall of 83.6%, with the best *F*_1_ score of 85.7% and the best accuracy of 93.9%. In contrast, CRF-based method [6] and HMM-based method [4] achieved significantly lower precision, recall and *F*_1_ scores. These results revealed that the high level feature stably achieved by the convex optimization regularized by mix-norm with nonnegative constraint has high discriminative ability and is essential for mitotic cell modeling. Consequently, the proposed method can outperform both methods even though no temporal context is incorporated. Because HCRF and EDCRF can capture the intermediate structures using hidden-state variables and is more flexible to model the temporal state transition, the HCRF-based method [5] and the EDCRF-based method [13] obtained better result in terms of *F*_1_ score (87.0% and 87.4%, resp.). However, both sacrificed high computation complexity only with 1.3% and 1.7% improvement of *F*_1_ score.

## 6. Conclusion

 In this paper, we propose a nonnegative mix-norm convex optimization method for mitotic cell detection. This method can overcome the difficulty in feature formulation for deformable objects. Moreover, it is independent of tracking or temporal inference model. Large scale comparison experiments demonstrate that the proposed method can produce competing results with the state-of-the-art methods by the highest *F*_1_ score (85.7%) and accuracy (93.9%). We plan to discover more characteristics of mitosis event with the biology knowledge for objective function design to improve the performance of mitotic cell detection.

## Figures and Tables

**Algorithm 1:**
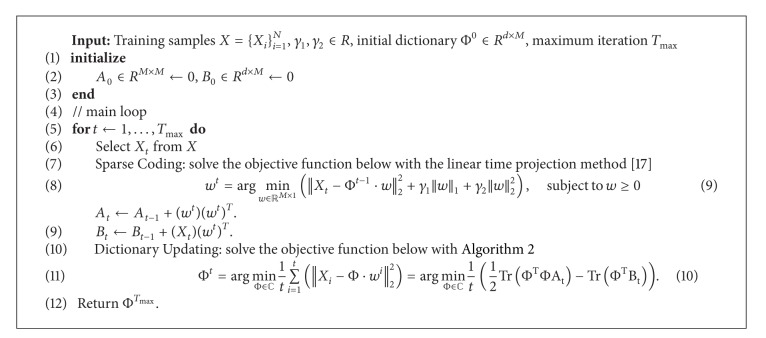
Iterative updating method.

**Algorithm 2:**
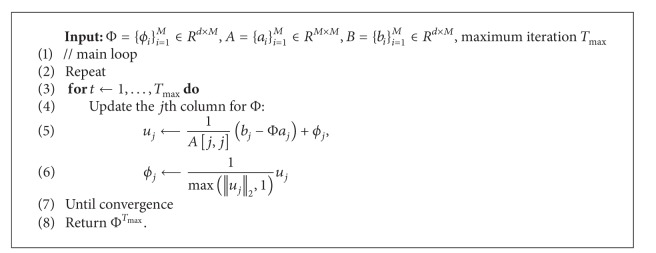
Dictionary updating algorithm.

**Table 1:** Performance comparison for mitotic cell detection with respect to four kinds of visual features (*γ*_1_ = 0.1 and *γ*_2_ = 0.1).

Criteria	Proposed (%)				SSM [1] (%)			
	Raw	SIFT	GIST	HoG	Raw	SIFT	GIST	HoG
Precision	88.0	76.9	87.7	65.7	70.1	64.3	65.0	55.5
Recall	83.6	86.1	83.6	86.1	77.2	88.9	92.2	75.5
*F*_1_ score	85.7	81.2	85.6	74.6	73.5	74.6	76.3	64.0
Accuracy	93.9	91.2	93.8	87.0	87.7	86.7	87.3	81.2

**Table 2:** Comparison of mitosis detection.

Model	Precision (%)	Recall (%)	*F*_1_ (%)
EDCRF [13]	89.2	85.7	87.4
HCRF [5]	83.0	90.0	87.0
CRF [6]	90.3	73.1	80.8
HMM [4]	82.0	77.2	79.5
Proposed	88.0	83.6	85.7
